# Pseudoxanthoma Elasticum Papillary Dermal Elastolysis: A Case Report

**DOI:** 10.1155/2010/352724

**Published:** 2010-08-16

**Authors:** Rubina Alves, Lurdes Ferreira, Esmeralda Vale, Olívia Bordalo

**Affiliations:** ^1^Department of Dermatology, Hospital Central of Funchal, Estrada dos Marmeleiros Monte, 9054-535 Funchal, Madeira, Portugal; ^2^Department of Dermatology, Centro Médico-Cirúrgico de Lisboa, Rua José Estevão, 35, 1600-100 Lisboa, Portugal

## Abstract

PXE-PDE is a rare clinicopathological entity with few cases reported. It affects more often elderly women and is characterized by asymptomatic bilateral and symmetrical yellowish papules localized predominantly on the neck and supraclavicular regions. It is clinically similar to Pseudoxanthoma Elasticum. The authors report a case of a 64-year-old woman presenting asymptomatic, yellowish, non-follicular papules, affecting the occipital and the posterior region of the neck for 1 year. The patient denied pruritic or inflammatory changes, marked solar exposition or trauma on the affected areas. Routine laboratory studies: thoracic x-ray and ophthalmologic examination were normal. The histopathologic examination of a biopsy of one of the cutaneous lesions showed an absence of elastic fibers in the papillary dermis.The diagnosis of Pseudoxanthoma Elasticum—like Papillary Dermal Elastolysis (PXE-PDE) was made. Of great importance is the differential diagnosis with Pseudoxanthoma elasticum (PXE), but we have also to consider other elastolytic disorders: mid-dermal elastolysis (MDE), linear focal elastosis (LFE) and white fibrous papulosis of the neck (WFPN). Until know, there is no effective treatment for this pathology.

## 1. Introduction

Pseudoxanthoma Elasticum Papillary Dermal Elastolysis (PXE-PDE) is a clinicopathological entity described for the first time by Rongioletti and Rebora in 1992. There are few cases reported. It affects more often elderly women and is characterized by asymptomatic bilateral and symmetrical yellowish papules localized predominantly on the neck and supraclavicular regions. It is dermatologically similar to Pseudoxanthoma Elasticum (PXE).

The absence or marked loss of elastic fibers on the papillary dermis and the absence of calcifications or fragmentation of the elastic fibers are characteristic of PXE-PDE. The etiopathogenic factors considered are intrinsic skin aging and ultraviolet radiation. Similar clinical and histopathological features have been also described in White fibrous papulosis of the neck and the designation of Fibroelastolytic papulosis of the neck (WFPN) was proposed encompassing the spectrum of the two diseases.

## 2. Clinical History

**Figure 1:**
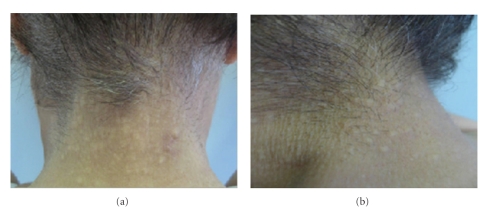
Clinical images of yellowish, symmetrical, and bilateral nonfollicular papules, located on the posterior and lateral region of the neck, respectively.

A 64-year-old caucasian woman presented in our department with one year slowly progressive appearance of nonfollicular papules, asymptomatic, located on the neck. The physical examination of the skin revealed the presence of multiple skin-colored and yellowish papules, with a cobblestone appearance, sized 1–6 mm, symmetrically distributed on the occipital, lateral, and posterior region of the neck (Figure 1). Cutaneous examination showed no other alterations.

The patient denied history of pruritic or inflammatory changes, marked solar exposition or trauma on the affected areas. She also denied topical and systemic drug use. There was no personal or family history of similar lesions.

She performed several diagnostic tests such as routine laboratory studies and thoracic X-ray, that were normal. Cardiac and ophthalmologic examination did not reveal any abnormalities.

**Figure 2:**
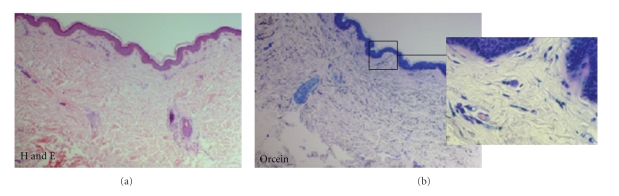
The histopathologic examination of a biopsy of one of the cutaneous lesions revealed: H&E stain: no alterations; Orcein stain: absence of elastic fibers in the papillary dermis.

We performed a biopsy of one of the cutaneous lesion of the posterior region of the neck for histopathologic examination. The hematoxylin-eosin stain (H&E) did not appear to reveal any alterations The orcein stain showed an absence of elastic fibers in the papillary dermis; the elastic component of the reticular dermis was normal (Figure 2). No calcifications were observed on Von Kossa stain.

Based on clinical history and histological examination, the diagnosis of pseudoxanthoma elasticum-like papillary dermal elastolysis (PXE-PDE) was made. No therapeutic measures were taken once the patient was asymptomatic.

## 3. Discussion

**Figure 3:**
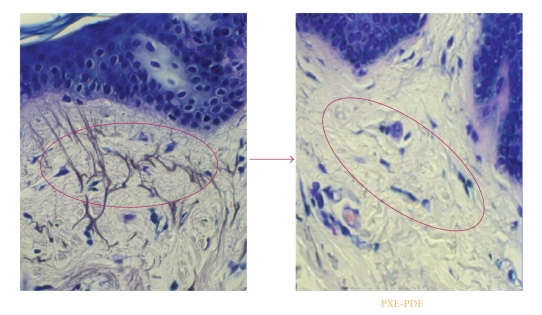
Presence of the elastic fibers in a perpendicular orientation on the dermis papillary (Normal) versus the absence of elastic fibers, presented in the PXE-PDE.

The elasticity of the skin is based on the structure of elastic fibers. In the papillary dermis, the oxytalan and elaunin fibers insert into the basement membrane in a perpendicular orientation and extend into the dermis (Figure 3), where they gradually merge with the elastic fibers that form a plexus parallel to the dermal-epidermal junction [1]. Dermis is a complex organ because, depending of the zone affected it seems to be under different controls. 

Our case is a report of an abnormality of the elastic fibers: pseudoxanthoma elasticum-like papillary dermal elastolysis (PXE-PDE). This disease affects more often elderly women and is characterized by asymptomatic and symmetrical yellowish papules localized predominantly on the neck, supraclavicular regions, and flexural areas [2–4].

The etiopathogenic factors are still unknown but some authors think it can be related with intrinsic skin aging and ultraviolet radiation [2, 5–8]. There is one familial case documented in the literature, suggesting an influence of genetic or inheritable factor [9].

The absence or marked loss of elastic fibers on the papillary dermis and the absence of calcifications or fragmentation of the elastic fibers are characteristic of PXE-PDE [5]. The collagen fibers are normal. In the immunohistochemistry studies, there are loss of elastin and fibrilin-1 [6, 10]. On aging, there is only loss of fibrilin-1 and elastin remains normal or decreased [10].

Of great importance is the differential diagnosis with Pseudoxanthoma elasticum (PXE), but we also have to consider other elastolytic disorders: mid-dermal elastolysis (MDE), linear focal elastosis (LFE), and white fibrous papulosis of the neck (WFPN).

Until now, there is no effective treatment for this pathology.

The PXE is a rare genetic disorder, caused by a mutation in the ABCC6 gene [11, 12]. The cutaneous lesions are similar in the two pathologies. The main findings of this disease are located in the mid and lower dermis and consist of fragmentation, clumping, and calcification of the elastic fibers [4, 13].

Unlike PXE-PDE, PXE usually develop during childhood, has systemic involvement, characterized by calcification of the elastic fibers of the skin, retina (angioid streaks), and cardiovascular system, which can lead to serious complications.

The mid-dermal elastolysis (MDE) is a rare disorder with clinical features of fine wrinkles located mostly on the trunk, lateral neck, and upper extremities.

Pathogenesis is still unknown, but some cases appear to be induced or aggravated by ultraviolet light exposure [14]. In the MDE, the histological changes correlate to a loss of elastic tissue in the mid dermis whereas the elastic tissue of the papillary and deep dermis remains apparently normal [13–17]. The linear focal elastosis (LFE) is an uncommon dermal elastosis that affects more frequently elderly men. Clinically the lesions appear as asymptomatic yellow striae-like bands, palpable, disposed horizontally on the lower back, its predominant location. Histologically, these lesions present with an increased number of clumped and fragmented elastic fibers in the mid and deep dermis separated by normal collagen [4, 18].

The white fibrous papulosis of the neck (WFPN) is an entity characterized by the presence of multiple confluent whitish papules, nonfollicular, mostly located on the neck. They resemble clinically PXE-PDE. The main pathologic feature of WFPN is thickened papillary dermal collagen with decreased elastic fibers [3, 8, 19].

Some authors consider there are some clinical and histopathological features between WFPN and PXE-PDE and propose the designation of Fibroelastolytic Papulosis of the neck, encompassing the spectrum of the two diseases [3, 8, 19]. Recently, Wang et al. [20], proposed a new benign elastic tissue disorder: papillary dermal elastolysis (PDE). Based on the authors, this entity resembles PXE-PDE and is characterized by the presence of focal clumps of elastic fibers alternating with areas of lack of oxytalan and eulanin fibers [20].

Perhaps in the future, we could englobe these three entities (WFPN, PXE-PDE and PDE) as Fibroelastolytic Papulosis.
